# Substance abuse prevalence and its relation to scholastic achievement and sport factors: an analysis among adolescents of the Herzegovina–Neretva Canton in Bosnia and Herzegovina

**DOI:** 10.1186/1471-2458-12-274

**Published:** 2012-04-05

**Authors:** Damir Sekulic, Marko Ostojic, Zdenko Ostojic, Braco Hajdarevic, Ljerka Ostojic

**Affiliations:** 1 Faculty of Kinesiology, University of Split, Teslina 6, Split, 21000, Croatia; 2 NIHON doo, Spinutska 65, Split, 21000, Croatia; 3 University Clinical Hospital Mostar, Kralja Tvrtka bb, Mostar, 63000, Bosnia and Herzegovina; 4 Regional Medical Center Mostar, Marsala Tita 294, Mostar, 63000, Bosnia and Herzegovina; 5 School of Medicine, University of Mostar, Bijeli brijeg, Mostar, 63000, Bosnia and Herzegovina

**Keywords:** Substance misuse, Drugs, Physical activity, Education

## Abstract

**Background:**

Substance abuse among adolescents is a major public health and social problem. However, studies rarely investigate the relationships between substance abuse, educational achievement and sport factors. Substance abuse is an even more significant problem in societies that have experienced trauma, such as Bosnia and Herzegovina, which have had recent wars. The aims of this study were to investigate substance abuse among adolescents in Bosnia and Herzegovina and to study the potential gender-specific relationships between a) sport factors (physical activity/exercise/athletic participation) and substance abuse and b) scholastic achievement and substance abuse.

**Methods:**

Our sample consisted of 1,032 adolescents who were 17 to 18 years old (435 boys and 597 girls) and who were in the final grade of high school. These subjects were randomly selected from the territory of Herzegovina-Neretva Canton of Bosnia and Herzegovina. Retrospective testing was performed using an extensive self-administered questionnaire. The questionnaire included questions involving topics such as sociodemographic variables, scholastic variables, sport factors, and substance abuse data (smoking habits, drugs consumption and alcohol consumption using the AUDIT questionnaire). Descriptive statistics, frequencies, analyses of the differences and correlational analyses were performed.

**Results:**

Our results found that greater than one-third of the boys and one-fourth of the girls were daily smokers, and almost half of the boys and one-fifth of the girls practiced harmful drinking; other drugs (i.e. heroin, cocaine, amphetamines, etc.) were rarely consumed. Boys dominated in sport factors, whereas girls were more successful in scholastic achievement. Approximately 23% of the boys and 6% of the girls reported that they practiced harmful drinking and smoked simultaneously. Educational failure, which was defined as having one or more negative grades at the end of the last two school years, was identified in 20% of the boys and 9% of the girls. In both genders, substance abuse was negatively correlated with educational achievement, and half of those students who failed educationally reported daily smoking. Among the girls who experienced education failure, 33% were smokers, and 22% practiced harmful drinking. Sport factors were weakly correlated with substance abuse in boys; thus, we could not support the hypothesis that sports are a protective factor against substance abuse among male adolescents. In girls, participation in team sports was related with a higher incidence of smoking, but there was no evidence of sport factors having an influence on the consumption of alcohol.

**Conclusion:**

In this study, the incidence of smoking and the consumption of alcohol were alarmingly high. These findings demonstrate the need for intervention programs to address these issues. These problems are particularly important, considering that substance abuse has a negative impact on educational achievement among boys and girls, and sport factors have not been found to be protective factors against substance abuse.

## Background

Adolescent substance abuse (SA), which includes the consumption of alcohol, cigarette smoking, the consumption of drugs and other behaviors, is a significant public health issue in the world today. In addition to being a serious, health-threatening behavior [1-3], SA is often associated with detrimental consequences and creates certain difficulties for not only the individuals who misuse the substances but also their parents, families, school, peers, and society as a whole [4-6]. Today, it is generally accepted that the earlier a child begins to use substances, the greater the chance that the child will become addicted [7,8]. In contrast, those who reach the age of 21 without smoking, consuming illegal drugs, or binge drinking are likely to never engage in these behaviors [9]. Therefore, it is particularly important to find any possible protective factors or risk factors for SA among adolescents.

The importance of physical activity is well known. Physical activity helps to reduce the risk of a number of critical health problems, including obesity, heart disease, stroke, colon cancer, diabetes and osteoporosis [10,11]. Additionally, participation in physical activity and sports among young people has been shown to promote social well-being, physical and mental health, academic achievement, self-discipline, and socialization [12]. It is also hypothesized that participation in sports and physical exercise will reduce the tendency of young people to abuse substances [13-17]. However, the literature to date has not consistently validated the perception that sport factors (i.e., physical activity, exercise, and athletic participation) are factors which could buffer SA among children and adolescents. Generally, there have been conflicting findings regarding the relationship between sport factors and the use of alcohol, tobacco, and other drugs. While some investigations have indicated a decreased likelihood of SA in sport participants [18,19], other studies have shown that sports and physical activities were associated with an increased likelihood of SA [20,21].. In some cases [22], authors have identified gender-specific relationships between sport participation and SA (i.e., an increased tendency toward SA in girls who participated in sports, but protective effects of sports against SA among boys of the same age). Additionally, in the later study [22], the authors concluded that some indices indicated that the participation in different types of sports (e.g., individual vs. team sports) may be an important issue when studying the association of sports and SA.

In Bosnia and Herzegovina (B&H), findings regarding SA among adolescents are limited, and few papers have investigated SA within this country. Studies that have focused on tobacco smoking [23,24] have reported a very high smoking incidence among the overall population (approximately 50% of men and 30% of women were daily smokers). In terms of alcohol consumption, the authors highlighted a high risk for alcoholism in the Mostar region, with 9.9% of the overall population, 11.1% of college-age students, and 14.4% of the high-school population being at risk [25-27]. An alarmingly high incidence of SA has been explained by the fact that B&H is a society that has experienced past trauma. It is likely that the country still suffers from the effects of a recent war that caused great material and social damage and resulted in an increase in SA among the overall population. However, it is clear that data demonstrates the need for an investigation evaluating SA problems and their consequences in the territory of B&H, especially among adolescents.

Because policy and prevention strategies against SA as a serious public health problem should rely on accurate data for each country, territory, culture, sociodemographic circumstances, and so on, we have determined that the problem of SA are particularly important. Therefore, the aims of this study were to investigate SA among adolescents in B&H and to assess the potential gender-specific relationships between sport factors, scholastic achievement, and SA among 17- to 18-year-old adolescents from B&H. Due to the well-known cultural and religious differences that exist in B&H, we wanted to study a relatively homogenous sample of subjects. Thus, we only sampled ethnic Croats from the Herzegovina-Neretva Canton (HNC).

## Materials and methods

In this study, the sample consisted of 1,032 17- to 18-year-old adolescents (435 boys and 597 girls) who were randomly selected from the four towns of Siroki Brijeg, Grude, Capljina and Posusje. All subjects were in their final grade of high school and were recruited randomly from the population of adolescents in the HNC who were in their final grade of middle school. We have used simple random sampling. By means of lottery method we have chosen (A) approximately one third of high schools in the HNC, and (B) one school shift (note that high schools in the HNC are mostly organized throughout two shifts). The testing was performed in one occasion, which means that pupils who were absent from school during the testing-day of testing were not included in the study. Consequently, the sample should be representative of the 10.5%of the 10.639 middle school-aged children in the HNC, which is approximately 20% of high-school final-graders in the HNCTesting was performed using an extensive self-administered questionnaire that consisted of the following four groups of variables: sociodemographic variables, scholastic variables, sport factors, and SA data. These variables are reviewed in the following text.

### Sociodemographic data

The sociodemographic variables in this study included age and gender.

### Scholastic variables

Subjects were asked about their scholastic achievement (grade point average) over the last two years (a five-point scale ranging from 1, which is poor, to 5, which is excellent), unexcused school absence (number of non-excused absences measured in teaching hours; a five-point scale ranging from “less than 5 hours”, to “20 hours and more”), behavioral grade (a three-point scale, exemplary – fair - good), and overall school absence (a four-point scale ranging from “almost never”, to “often”)

### Sport factors

Subjects answered questions regarding their participation in sports and the results that they achieved in sports. Because previous studies have suggested that the participation in team and individual sports can have different effects on SA [28], we assessed individual and team sport participation separately. Additionally, because it is well known that most substances can impair sport performance [29], SA in adolescents might be influenced by the dedication of the adolescent to sports and exercise. Therefore, subjects were asked about their sport participation, the amount of time that they spend involved in sports and their sport achievements using an ordinal questionnaire form.

**Table 1:** Substance abuse (marihuana, hashish, LSD) among male and female adolescents (F – frequency, % - percentage) and Kruskal Wallis ANOVA between sexes (H – H value; p – level of significance)

	**BOYS**		**GIRLS**		**Kruskal Wallis**	
	**F**	**%**	**F**	**%**	**H**	**p**
**Marihuana**						
Never (1)	361	82.99	559	93.63		
Ever tried (2)	31	7.13	14	2.35		
Once or twice (3)	12	2.76	2	0.34		
3-5 times (4)	2	0.46	-	-		
6-9 times (5)	4	0.92	4	0.67		
20-39 times (6)	-	-	2	0.34		
40 and more (7)	12	2.76	1	0.17		
Missing	13	2.99	15	2.51	35.47	0.001
**Hashish**						
Never (1)	399	91.72	580	97.15		
Ever tried (2)	7	1.61	1	0.17		
Once or twice (3)	3	0.69	1	0.17		
3-5 times (4)	1	0.23	-	-		
6-9 times (5)	3	0.69	2	0.34		
20-39 times (6)	-	-	-	-		
40 and more (7)	6	1.38	-	-		
Missing	16	3.68	13	2.18	17.46	0.001
**LSD**						
Never (1)	410	94.25	580	97.15		
Ever tried (2)	3	0.69	-	-		
Once or twice (3)	-	-	-	-		
3-5 times (4)	-	-	-	-		
6-9 times (5)	3	0.69	1	0.17		
20-39 times (6)	1	0.23	-	-		
40 and more (7)	5	1.15	-	-		
Missing	13	2.99	16	2.68	13.65	0.001

LEGEND: number in parentheses presents ordinal value for each variable.

### SA data

Subjects were asked about their smoking habits, alcohol consumption, and drug (i.e., opiates) usage. Smoking was tested on a six-point scale that ranged from “never smoked” to “smoking more than a pack daily.” Alcohol consumption was measured using the AUDIT questionnaire [30,31]. On this questionnaire, subjects answers ten items and scores for each item ranged from 0 to 4, which defines the hypothetical range of minimally 0 to maximally 40 The results that focused on alcohol consumption were later divided into “harmful drinking” (scores of 11 or above) and “non-harmful drinking” (scores below 11) [32]. However, in the correlation analysis (see later text), the actual numerical result of each examinee was used. Although the AUDIT offers three different scores (consumption score, dependence score, and alcohol related problems) in our sample of subjects the overall AUDIT results were mostly influenced by questions related to “consumption score”. Therefore in this study the scores for each examinee were not separated but were observed as overall AUDIT result. The sub-scale for drug consumption included questions about the consumption of marijuana, hashish, heroin, cocaine, and most of the party drugs (e.g., ecstasy, amphetamines, and others). A seven-point range of consumption was offered for each question (ranging from “never” to “40 times and more”).

### Testing and ethical permissions

Testing was strictly anonymous, meaning that no personal data were collected (e.g., date of birth, city of birth, or specific club or sport participation). Multiple-choice answers were offered when possible (see Results for more details). Testing occurred in a group of at least 15 examinees. Each examinee was told that the testing was strictly anonymous, that he/she could refuse to participate and that he/she could leave some questions and/or the entire questionnaire unanswered. Each examinee received two questionnaire forms and one envelope. When testing was completed, each examinee placed one form (answered or unanswered) in the envelope and sealed it. He/she immediately destroyed the second form in a paper shredder. The next day, the envelopes were opened by an investigator who did not test the subjects. Before the study, the complete procedure and aim of the testing were explained to all subjects and at least one parent. Informed consent was obtained. The response rate was over 99%. The study fulfilled all ethical guidelines and received the approval of Ethical Board of School of medicine, University of Mostar.

In the first phase of statistical processing, all variables were assessed using the Kolmogorov-Smirnov test normality of the distribution to establish the variables’ parametric/nonparametric natures. Because the Kolmogorov-Smirnov test identified all of the variables (except AUDIT) as non-parametric, which is also known as ordinal, the descriptive statistics included the calculation of the counts and frequencies. Differences between genders were established using the Kruskal-Wallis test and a t-test for independent samples (for AUDIT). The relationship between and within sport factors, scholastic variables, and SA were examined using the Spearman rank-order correlation.

## Results

More than one-third of the boys and one-fourth of the girls were smokers. In our sample, opiates, marijuana and hashish were rarely consumed. However, approximately 6% of the boys consumed marijuana. Interestingly, party drugs, such as speed and ecstasy, were rarely consumed. Forty-seven percent of the boys and 18% of the girls practiced harmful drinking. In general, boys were more prone to abuse substances than girls. Because AUDIT was the only variable that the K-S test recognized as parametric in nature, the t-test that was calculated between genders revealed that boys were more prone to consuming alcohol than their female peers (Tables 1,2,3).

**Table 2:** Substance abuse (smoking, alcohol, ephedrine, speed and ecstasy) among male and female adolescents (F – frequency, % - percentage), Kruskal Wallis ANOVA and T-test between sexes (H – H value; t – t value; p – level of significance)

	**BOYS**		**GIRLS**		**Kruskal Wallis** **(T Test)**	
	**F**	**%**	**F**	**%**	**H (t)**	**p**
**Smoking…**						
I have never smoked (1)	167	38.39	324	54.27		
Quit smoking (2)	15	3.45	30	5.03		
Occasionally, from time to time, but not daily (3)	92	21.15	139	23.28		
Less than 10 cigarettes per day (4)	106	24.37	72	12.06		
10-20 cigarettes per day (5)	46	10.57	22	3.69		
More than a pack per day (6)						
Missing	9	2.07	10	1.68	44.69	0.001
Alcohol drinking (AUDIT) *						
Non-harmful drinking	224	-	482			
Harmful drinking	204	-	105			
Missing	7	1.61	10	1.67	12.04	0.001
**Ephedrine**						
Never (1)	415	95.40	581	97.32		
Ever tried (2)	2	0.46				
Once or twice (3)	1	0.23	1	0.17		
3-5 time (4)	2	0.46				
6-9 times (5)						
10-19 times (6)	1	0.23				
Missing	14	3.22	15	2.51	5.53	0.020
**Cocaine**						
Never (1)	406	93.33	580	97.15		
Ever tried (2)	5	1.15	-	-		
Once or twice (3)	3	0.69	1	0.17		
3-5 times (4)	1	0.23	-	-		
6-9 times (5)	2	0.46	-	-		
10-19 times (6)	5	1.15	-	-		
Missing	13	2.99	16	2.68	19.21	0.001
**Speed and Ecstasy**						
Never (1)	404	92.87	578	96.82		
Ever tried (2)	6	1.38	2	0.34		
Once or twice (3)	4	0.92	1	0.17		
3-5 times (4)	2	0.46	-	-		
6-9 times (5)	3	0.69	-	-		
20-39 times (6)	-	-	-	-		
40 and more (7)	3	0.69	-	-		
Missing	13	2.99	16	2.68	16.84	0.001

LEGEND: * Differences between genders for AUDIT were calculated by t-test; number in parentheses presents ordinal value for each variable.

**Table 3:** Substance abuse (heroin, sedatives, inhalants) among male and female adolescents (F – frequency, % - percentage) and Kruskal Wallis ANOVA between sexes (H – H value; p – level of significance)

	**BOYS**		**GIRLS**		**Kruskal Wallis**	
	**F**	**%**	**F**	**%**	**H**	**p**
**Heroin**						
Never (1)	411	94.48	583	97.65		
Ever tried (2)	1	0.23	-	-		
Once or twice (3)	2	0.46	1	0.17		
3-5 times (4)	-	-	-	-		
6-9 times (5)	-	-	-	-		
20-39 times (6)	3	0.69	-	-		
40 and more (7)	4	0.92	-	-		
Missing	14	3.22	13	2.18	10.99	0.001
**Sedatives**						
Never (1)	400	91.95	545	91.29		
Ever tried (2)	9	2.07	25	4.19		
Once or twice (3)	-	-	6	1.01		
3-5 times (4)	1	0.23	-	-		
6-9 times (5)	4	0.92	1	0.17		
20-39 times (6)	3	0.69	3	0.50		
40 and more (7)	3	0.69	3	0.50		
Missing	15	3.45	14	2.35	1.25	0.240
**Inhalants**						
Never (1)	390	89.66	555	92.96		
Ever tried (2)	6	1.38	18	3.02		
Once or twice (3)	10	2.30	5	0.84		
3-5 times (4)	1	0.23	2	0.34		
6-9 times (5)	6	1.38	-	-		
20-39 times (6)	-	-	1	0.17		
40 and more (7)	9	2.07	1	0.17		
Missing	13	2.99	15	2.51	4.35	0.037

LEGEND: number in parentheses presents ordinal value for each variable.

Girls were more successful in educational achievements, and significant differences were identified between sexes in all four of the scholastic variables (Table 4).

**Table 4:** Scholastic achievement variables among male and female adolescents (F – frequency, % - percentage) and Kruskal Wallis ANOVA between sexes (H – H value; p – level of significance)

	**BOYS**		**GIRLS**		**Kruskal Wallis**	
	**F**	**%**	**F**	**%**	**H**	**p**
**Scholastic achievement**						
excellent (5)	86	19.77	56	9.38		
very good (4)	20	4.60	10	1.68		
good - average (3)	174	40.00	145	24.29		
sufficient – bellow average (2)	119	27.36	248	41.54		
insufficient (1)	36	8.28	135	22.61		
Missing	0	0.00	3	0.50	89.40	0.001
**School absence…**						
almost never (4)	108	24.83	210	35.18		
rarely (3)	163	37.47	233	39.03		
from time to time (2)	125	28.74	133	22.28		
often (1)	37	8.51	18	3.02		
Missing	2	0.46	3	0.50	22.91	0.001
**Unexcused school absence…**						
< 5 (5)	213	48.97	462	77.39		
5-10 (4)	111	25.52	89	14.91		
11-15 (3)	59	13.56	17	2.85		
16-20 (2)	42	9.66	7	1.17		
20 ≥ (1)	8	1.84	10	1.68		
Missing	2	0.46	12	2.01	108.84	0.001
**Behavior grade…**						
exemplary (3)	255	58.62	505	84.59		
fair (2)	112	25.75	76	12.73		
poor (1)	67	15.40	13	2.18		
Missing	1	0.23	3	0.50	97.65	0.001

LEGEND: number in parentheses presents ordinal value for each variable.

Boys dominated in all of the sport factors that we studied. Briefly, they were more involved in individual and team sports, they practiced sports for a longer amount of time, and they achieved a higher competitive status (result) than girls (Table 5).

**Table 5:** Sport factors among male and female adolescents (F – frequency, % - percentage) and Kruskal Wallis ANOVA between sexes (H – H value; p – level of significance)

	**BOYS**		**GIRLS**		**Kruskal Wallis**	
	**F**	**%**	**F**	**%**	**H**	**p**
**Individual sport participation…**						
Yes, I’m still participating (1)	118	27.13	58	9.72		
Yes, but not anymore (2)	166	38.16	153	25.63		
No, never (3)	149	34.25	380	63.65		
Missing	2	0.46	6	1.01	99.72	0.001
**Team sport participation**						
Yes, I’m still participating (1)	152	34.94	62	10.39		
Yes, but not anymore (2)	193	44.37	215	36.01		
No, never (3)	87	20.00	314	52.60		
Missing	3	0.69	6	1.01	145.74	0.001
**Time of the sport involvement**						
Never involved in sport (1)	61	14.02	267	44.72		
< 1 year (2)	66	15.17	90	15.08		
1-5 years (3)	111	25.52	154	25.80		
5 ≥ years (4)	194	44.60	82	13.74		
Missing	3	0.69	4	0.67	153.77	0.001
**Sport competitive achievement…**						
I have never competed (1)	137	31.49	383	64.15		
Competition of the lower ranks (2)	200	45.98	135	22.61		
Competition of the national level(3)	70	16.09	39	6.53		
National team (4)	24	5.52	30	5.03		
Missing	4	0.92	10	1.68	95.21	0.001

LEGEND: number in parentheses presents ordinal value for each variable.

In both genders, SA was negatively correlated with most of the scholastic variables (Table 6).

**Table 6:** Correlation analysis between cigarette smoking and Alcohol consumption (AUDIT) with scholastic factors and sport factors (* denotes significant coefficients)

	**Smoking**		**AUDIT**	
	**Boys**	**Girls**	**Boys**	**Girls**
Scholastic achievement	−0.23*	−0.28*	−0.09	−0.05
School absence	−0.26*	−0.29*	0.23*	0.22*
Unexcused school absence	−0.33*	−0.33*	0.33*	0.27*
Behavior grade	−0.34*	−0.36*	0.37*	0.26*
Individual sport participation	0.03	0.00	−0.04	−0.03
Team sport participation	0.23*	0.03	−0.02	0.01
Time of the sport involvement	−0.13*	−0.01	0.07	0.05
Sport competitive achievement	−0.13*	0.02	0.09	0.03

The sport factors (i.e., time of sport involvement and competitive status) results were significantly but numerically low and were negatively correlated with smoking habits. No significant correlation between SA and sport factors was identified among the girls (Table 6).

**Figure 1:**
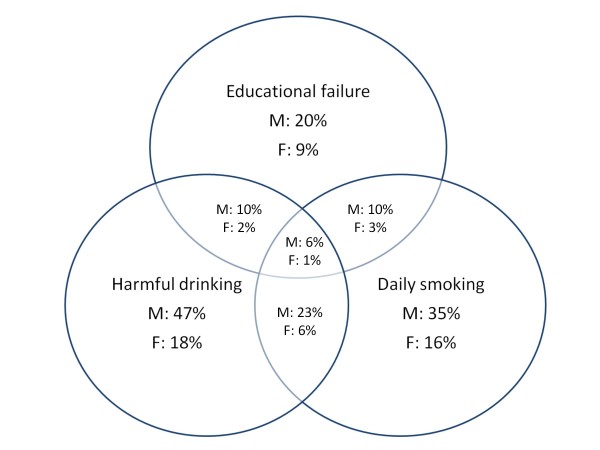
**Concomitant self-reported current involvement in individual sports, daily tobacco smoking and harmful drinking.** Percentage of reported frequency for boys (M) girls (F) with n = 435 and n = 597, respectively, being the 100%.

**Figure 2:**
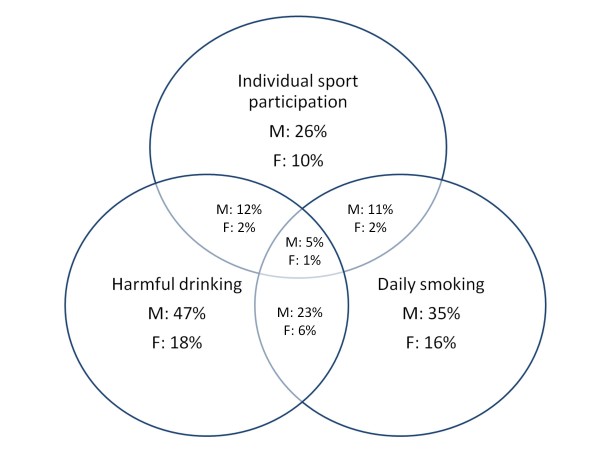
**Concomitant self-reported current involvement in team sports, daily tobacco smoking and harmful drinking.** Percentage of reported frequency for boys (M) girls (F) with n = 435 and n = 597, respectively, being the 100%.

**Figure 3:**
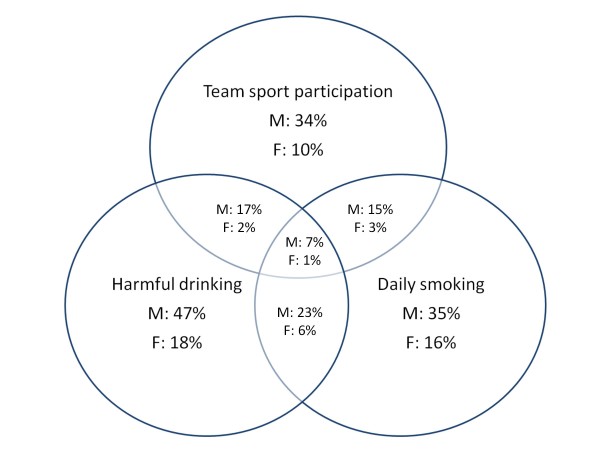
**Concomitant self-reported educational failure, daily tobacco smoking and harmful drinking.** Percentage of reported frequency for boys (M) girls (F) with n = 435 and n = 597, respectively, being the 100%.

Because of the low correlation coefficients, we used current participation in individual and team sports, daily smoking and harmful alcohol consumption to determine the associations between sport factors and SA. The same approach was used to determine the relationships between educational failure, daily smoking and harmful alcohol consumption (Figure 1, 2 and 3).

Approximately 23% of the boys and 6% of the girls practiced harmful drinking simultaneously to daily smoking. Educational failure (one or more negative grades at the end of the last two school years) was found in 20% of the boys and 9% of the girls. There was a clear relationship between educational failure and smoking in both genders, as half of those students who failed educationally reported daily smoking. A similar trend was found between harmful drinking and educational failure for boys, as 50% of the boys who failed educationally also practiced harmful drinking. Among the girls who failed educationally, 33% were smokers, and 22% practiced harmful drinking. However, for a more precise analysis, we compared these percentages to the proportion of the overall sample (see Discussion). Educational failure, together with harmful drinking and daily smoking, was identified among 7.1% of the boys and 1.6% of the girls.

Half of the boys and 20% of the girls who were involved in team sports practiced harmful drinking, while 44% of the boys and 30% of the girls who were involved in team sports were daily smokers. Of those who were involved in individual sports, the results were different. Forty-six percent of the boys and 20% of the girls who were involved in individual sports participated in harmful drinking, and 42% of the boys and 20% of the girls who were involved in individual sports smoked cigarettes on a daily basis.

## Discussion

### Substance use and misuse among adolescents from Bosnia and Herzegovina

The high incidence of smoking puts B&H among the countries with the highest smoking incidence in Europe. Vasilj et al. [23] reported data from the Non-Communicable Risk Factors Survey from 2002, in which 49.2% of men and 29.7% of women reported smoking on a daily basis. This is similar to data presented by Pilav et al. [24]. Consequently, the high frequency of adolescent smoking in our study (35% of the boys and 16% of the girls smoked cigarettes daily) can be viewed as the result of the high smoking prevalence in the general population. In the territory of HNC, from which the sample was drawn, the problem of smoking is almost certainly influenced by a specific cultural heritage. The HNC is historically known as a region where tobacco growing has been an important part of the economy since the 17^th^ century. This has contributed to the overall perception and acceptance of tobacco smoking in society. According to the most recent report of the European School Survey Project on Alcohol and Other Drugs (ESPAD) [33], the highest proportions of cigarette use during the last 30 days prior to the survey were identified in adolescents from Austria (42% and 48% for boys and girls, respectively), the Czech Republic (44% and 35% for boys and girls, respectively), Latvia (44% and 39% for boys and girls, respectively), Bulgaria (36% and 44% for boys and girls, respectively), and Croatia (38% and 38% of boys and girls, respectively). It must be emphasized that the ESPAD results relate to “monthly smoking,” while the results that we have presented denote “daily smoking habits,” and, therefore, are clearly alarming. In a recent study [22], authors examined SA among Croatian adolescents and when compared to our data smoking incidence for boys is similar to those we have presented. At the same time, the incidence of smoking is evidently higher among Croatian girls than among B&H girls. In their study [22] , there was no clear difference between genders for smoking, which is different from our findings that boys smoked more than girls.

The problem of alcohol consumption is known to be culturally specific [34,35]. Because of the relatively systematic sampling procedure (see Methods), we believe that our study design allowed us to reliably determine the practice of alcohol consumption among the adolescents for the territory of HNC. Generally, there is limited data available on alcohol use and misuse in the territory of B&H. In addition to a study performed by Skobic et al. [27], which we reviewed in the Introduction, we could not find any additional papers or reliable reports on the drinking behaviors of adolescents in B&H. Data we have presented for drinking behaviors are quite similar to those recently reported for Croatian adolescents [22], which measured significantly higher alcohol consumption in boys than in girls. This finding is in agreement with recent studies performed on a student population [36]. However, our finding that 47% of boys practiced “harmful drinking” is a highly alarming result; although drinking patterns among girls are significantly lower, the occurrence of harmful drinking in one of five girls is also highly disturbing, especially when compared to the European Union (EU). The most recent ESPAD data [33] reported that Denmark has the highest proportion of binge drinkers in the EU, with 49% of 16-year-old students (51% boys and 47% girls) having been drunk during the last 30 days. Because problem drinking during adolescence is associated with problem drinking in early adulthood [37], these incidences demonstrate the need for a serious intervention program among adolescents from B&H.

Opiates and cannabinoids were rarely used among the B&H adolescents that we evaluated. Most probably, traditional orientation within the society (see later text where we will discuss it in more details) should be considered as the most important barrier against consumption of drugs like heroin, cocaine, amphetamines, etc. Marijuana and hashish were the only drugs worth reporting, and only 1-2% of boys and less than 1% of girls were serious consumers (i.e., smoked marijuana and/or hashish more than 20 times in their lifetimes). This incidence puts adolescents from the HNC among the European countries with the lowest incidences of this habit; according to ESPAD, it is within the bottom 10^th^ percentile [33]. Lifetime use for boys in the HNC was similar to Malta (15%), Iceland (10%) and Greece (10%); for girls, lifetime use was similar to Greece (3%), Cyprus (3%) and Romania (2%).

### Substances and scholastic achievement

As previous studies have noted, it is difficult to define a causal relationship between scholastic variables and SA [38]. One of the main problems in defining this relationship is that it is not clear if SA causes educational failure or if educational failure leads to SA (i.e., those who experience educational failure are more inclined to gravitate toward sub-cultures where SA is more frequent; therefore, they start to use substances as a result of scholastic failure). A probable reason for the lack of clarity in this relationship is likely related to the lack of consistency in the educational factors (scholastic variables) that relate SA and scholastic achievement that are examined in studies. For example, some studies have used variables of academic achievement (e.g., grades and test scores), some focused on motivation for further education (e.g., college plans), and some studies included “school dropout” as a factor of interest. Interestingly, very few studies examined different scholastic variables in relation to substance use and misuse. It must also be noted that different educational systems consider different variables to be measures of school success for students. In the territory of the former Yugoslavia, school success is generally measured through three main categories: grade points (a five-point scale ranging from 1, which is poor, to 5, which is excellent in our case), school attendance (number of non-excused absences measured in teaching hours), and behavioral grade (three point scale). Although the proportion of overall school attendance is not officially considered a factor in student scholastic achievement, we included it as a variable of interest because of its presumed relationship with SA (see Results). Out of all of the substances, cigarettes have the strongest relationship with scholastic variables, including school-related problems [39,40]. In our study, although correlation coefficients were small, smoking was negatively correlated with scholastic achievement in both genders. Interestingly, the proportion of the explained variance when calculating relationships between variables was practically equal in boys and girls. Additionally, in both sexes, the smallest correlation between smoking habit and educational variables was identified when relating cigarettes and grade point average. However, because the correlation analysis only revealed a general relationship between the variables, a more specific description of the ratio between educational failure (one or more negative grades at the end of the previous academic year) and smoking habits is presented in Figure 1. It seemed that most of the boys who failed educationally were regular smokers. When the percentage of those who both failed educationally and were also smokers was compared to the percentage of smokers in the overall sample, there was a clear difference between these two percentages (50% and 35%, respectively). Among girls, that is even more apparent. Briefly, 16% of the girls were daily smokers, whereas within the sample of girls who failed educationally (9% of all girls), the proportion of smokers was much higher (30%). Consequently, the prevalence of smoking among those who failed educationally was much higher than in the overall sample (50% versus 35% for boys and 30% versus 16% for girls). Therefore, although the correlation analysis could lead us to conclude that the negative relationship between smoking and educational achievement is equal in both sexes, an additional analysis of comparisons of the proportions revealed that smoking should have more of a detrimental impact on scholastic failure in girls than in their male peers.

According to previous studies, it seems that the connection between alcohol use and educational variables varies across different ages and different sub-samples. In a study by Schulenberg et al. [41], the researchers found that college plans had an indirect positive effect on post-high school alcohol use. In another study, the authors found that alcohol consumption when subjects were in high school was positively related to their years of education at age 20 [42]. Accordingly, it seems that college attendance relates to a higher incidence of drinking during late adolescence. At the same time, other authors have identified a negative influence of alcohol consumption on educational achievement [43,44]; these findings were recently supported in one of the rare studies that investigated the problem of educational achievement in relation to alcohol consumption during adolescence in the territory of the former Yugoslavia. The authors who performed this study on adolescents in the former Yugoslavia reported consistent negative relationships between the AUDIT scores and all educational variables in Croatian adolescents of both sexes [22]. Educational achievement, which was determined by grade point average, was not significantly related to the AUDIT scores in either girls or boys. Data also showed similar percentages of those who practiced harmful drinking in the overall sample (47% among boys and 18% among girls) in comparison to the proportion of those who failed educationally and practiced harmful drinking (51% and 22% for boys and girls, respectively). Furthermore, a small but significant correlation between alcohol consumption and other scholastic variables (i.e., behavioral grade, unexcused school absence, and overall absence from school) showed that consuming alcohol was negatively correlated with these variables in adolescents from the HNC. Although we have not investigated the problem specifically, we will offer some possible explanations that should be investigated in the future. First, the high incidence of drinking in the general population (see previous text where drinking habits are discussed) is likely one of the reasons why the negative influence of drinking behavior is not as significantly related with school success as it was in previous studies in the HNC [22]. Alcohol consumption (especially traditionally breaded wines) is a widely accepted practice in the HNC. The prevalence of alcohol in the HNC is high, and recent studies found that 1.7% of alcohol addicts and 14.4% of people in the high school population who have a high risk of alcoholism reside in Mostar, the largest city in the HNC [27]. As a result, authors of that study underlined the need for extensive and urgent alcohol prevention strategies. Although we did not include a scale of alcohol addiction, the comparison of our data with data from the EU (see previous discussion) supports the conclusions of our respected colleagues that strategies to prevent alcohol consumption are necessary. The HNC is in a Mediterranean part of B&H and is therefore a region with a “Mediterranean style of drinking” (i.e., wine is consumed regularly during meals). Due to this high level of alcohol consumption in general and the social acceptance of drinking, alcohol consumption is most likely not highly related to school success among adolescents. However, we must note that our study did not include adolescents who are “out of the school system” (e.g., those who are suspended or expelled), which would likely influence the relationships we have discussed so far. As a result, alcohol consumption should be considered as a factor that has a relatively low negative association with scholastic achievement in the HNC.

### Sport participation in relation to substance use

The potential association of sport-physical activity and SA is particularly interesting during adolescence [14,45,46]. Our study is most likely the first one to examine the relationship between sport participation and SA among adolescents in B&H. In previous studies, authors have evaluated sports as a potential factor of influence on substance use and misuse through observing the intensity of physical activity during sports [28], leisure time and physical activity frequency [13], types of sport and physical activity participation [14]. However, recent investigations have highlighted the importance of “sport achievement” as a potential factor of influence on substance use in adult [29,47] and adolescent athletes [22]. Briefly, the rationale of such an approach is found in the fact that those who are systematically involved in sports will avoid substances that could diminish their athletic performance (i.e., cigarettes). Logically, if this is the case, then sport achievement will be negatively correlated with the consumption of such substances. At the same time, those substances that do not affect physical capacities, if taken at the right time, will be consumed more frequently, and the protective effect of sports against such substances (i.e., alcohol) will not be as evident. In this study, we have tried to take a step forward in the assessment with the inclusion of an analysis of the effect of team and individual sports on SA among adolescents. More precisely, we hypothesized that there will be a specific association with SA depending on whether team or individual sports are considered. The participation in team sports, achievement in sports and time of involvement in sports were determined to be weak factors negatively related to smoking habits among boys. At the same time, no significant correlation was noted for girls. However, more specific insight into the association between harmful drinking, everyday smoking (the most serious SA in our case) and sport participation showed that participation in individual sports should not be considered a buffering factor against SA in both sexes. Moreover, there were certain indices that showed that those who practiced sports were more often smokers and practiced harmful drinking. More precisely, if we related the 11% of daily smokers who were also involved in individual sports to the 26% of boys in the overall sample who practiced individual sports, there was a 38% incidence of daily smoking among those who were involved in individual sports. This is a slightly higher percentage than the 35% of daily smokers in the overall sample. The results were similar among girls (20% of the athletes were smokers compared to the 16% of the total female sample that were smokers). For alcohol, the situation was somewhat better, although the percentages of those who practiced harmful drinking and who were involved in individual sports was equal to the “drinkers” in the overall sample (46% versus 47% among boys and 20% versus 18% among girls). The relationship between team sport involvement and drinking habits demonstrated that sports have a negligible relationship with alcohol consumption. Briefly, 50% of the boys and 20% of the girls who were involved in team sports practiced harmful drinking, which is very similar to the 47% of boys and 18% of girls who had the same habit in the overall sample. The most disturbing fact that we found was that the proportion of boys who were daily smokers was higher for those who practiced team sports than for the percentage of boys who smoked daily in the overall sample (44% versus 35%). The situation was even more concerning among the girls in the sample (30% versus 16%).

In most cases, sport participation has been correlated with lower rates of cigarette usage [15-17], and such relationships are elegantly explained through the reasoning that smoking impairs respiratory function and consequently negatively influences physical capacities [48]. However, in our study, this was not the case. Sport participation (especially team sport participation) was determined to be a certain risk factor for smoking, especially among girls. Although not in accord with previous findings, from our point of view, such results can be explained by the sociocultural circumstances in the region from which the sample was drawn. The HNC is a region that is characterized by traditional gender roles, which should be highlighted as one of the important reasons for evident differences between genders in sport factors (see Results). Studies have already noted that traditional societies that observe traditional gender roles do not accept smoking among women [49,50]. Furthermore, there is a concern that women may take up smoking if rapid social change leads to an alteration in traditional gender norms that discourage this behavior. At the same time, it is also clear that traditionally strong gender roles are negatively related to female sport participation [51-53]. Therefore, although not assessed specifically herein, we may reason that sport participation among girls should be considered as an indicator of a more “liberal” and less traditional orientation. As a result, we believe that it is not the case that sport involvement increases SA in girls but rather that those girls who practice sports are more oriented toward the non-traditional roles of women, thus gravitating toward sports and substances simultaneously. To support this explanation, we highlight the situation among boys, in which there are equal proportions of smokers among boys who practice sports and boys in general.

The reason why a clear association between sport factors and alcohol consumption is not observed can be explained by the fact that in B&H, alcohol and sports are closely linked through sponsorships and advertising [54,55]. Many sporting organizations, teams, and events are supported by companies who make beer and other alcoholic drinks. An example of this is observed with the national basketball champions and the fact that a team from one of the towns that is included in this study is directly supported and named by a local beer manufacturer. While most athletes do not drink alcohol because they believe that it may improve their sports performance [56], alcohol consumption by athletes generally occurs in a social environment [57,58]. However, these social occasions are often embedded in the culture that surrounds participation in sports [59]. Binge-drinking episodes are frequently the topic of stories or media reports about the off-field exploits of athletes. These appear to occur most often after a session of sports, at sport-related social events, or in the company of other athletes. Post-exercise drinking is rationalized and justified by athletes in many ways, including “everyone is doing it,” “I only drink once a week” and “I can run/sauna it off the next morning.” In some cases, these episodes are romanticized, and the drinking prowess of the athletes is admired. The media covers such “incidents” extensively; consequently, adolescent athletes do not recognize drinking as harmful. Additionally, there is no doubt that in the region we have studied herein, alcohol consumption is socially acceptable (see previous discussion). Alcohol consumption is a habit that is as common in sports as it is in a non-sporting environment. Consequently, no clear differences between harmful drinking among adolescent athletes and the overall sample were identified.

### Study limitations

Several limitations should be considered with regards to study. First, the findings are based on subjects’ self-reports. Thus, it may be argued that subjects may not have told the truth if they felt uncomfortable. However, the strict anonymity of the questionnaire as well as the testing design (see Methods) decreased this possibility. The second limitation we have found in the fact that we actually tested adolescents who were “at school” and, therefore, certain data are practically missing (e.g., data of children who left the school). However, one the most important issue in this study was to define the relationship between sport factors, school success and SA in B&H adolescents. Since children who do not attend school practically cannot be included in organised sport, the selection of subjects likely did not significantly influence the results. Finally, we have conducted retrospective study and such design allows insights into associations between studied variables, but not into the causes and possible effects. Therefore in the following investigations, the intervention approach is needed. However, in spite of the study’s limitations, we believe that the results (although not the final word on the topic) contribute to knowledge in the field.

## Conclusion

Although the study was performed with a relatively homogenous sample of examinees from one canton in B&H, some specific findings need to be highlighted.

The rates of alcohol consumption and cigarette smoking demonstrate the need for an intervention strategy against the consumption of these substances among adolescents from the HNC. The incidence of cigarette smoking in B&H (35% of boys and 16% of girls are consuming cigarettes on a daily basis) is one of the highest reported prevalence levels among European countries. Alcohol consumption has also reached alarming rates, especially among adolescent males, as we identified harmful drinking behaviors among 50% of subjects tested. Both problems are likely related to the high occurrence of cigarette smoking and alcohol consumption throughout the country and to the social norms that find smoking and drinking acceptable. Smoking is not banned in open or closed facilities, such as nightclubs, bars and other public places, and age is not verified when cigarettes and alcohol are sold; additionally, the entire territory of the HNC is traditionally known for growing vines and tobacco. Of note, opiates, cannabinoids and party drugs (e.g., speed and ecstasy) were rarely used within the studied population.

Our analysis revealed that smoking is negatively correlated with educational achievement. However, the relationship between SA and education is more obvious among girls, likely due to the lower consumption rates of substances in this group. To a lesser extent, scholastic failures are also associated with alcohol consumption. Because the methodological approach that we have used does not allow us to define how SA influences educational failure or vice versa, we can highlight the clear need for an additional analysis of this problem in future studies.

The relationship that was identified between sport factors and SA revealed that sports should not be considered a factor which is negatively associated to SA among adolescents. Among boys, the relationship between sport factors and SA was negligible, but among girls, the data highlighted an increased tendency for smoking among those girls who were involved in team sports. Therefore, sports are not considered to be a risk factor for SA for girls, but rather those girls who are involved in sports are generally less traditionally oriented and thus gravitate toward substance use and sport participation simultaneously.

## Competing interests

The authors declare that they have no competing interests.

## Authors’ contributions

DS designed the study, performed statistical analysis and discussed data. MO collected the data, drafted the manuscript and discussed the results; ZO did preliminary statistical procedures and discussed the data; BH collected the data and did preliminary statistics, overviewed the previous researches; LO participated in the study design and drafted the manuscript. All authors have read and approved the final version.

## Pre-publication history

The pre-publication history for this paper can be accessed here:

http://www.biomedcentral.com/1471-2458/12/274/prepub
